# 

**Published:** 1908-01-15

**Authors:** 


CONTENTS

Event and Comment - - - - - - - - - - 1
Best Methods of Avoiding Unnecessary Display of Gold in Bridge-Work, - - - - - *By W. A. Giffen, D.D.S.* 5
Cases of Oral Surgery, - *By Charles H. Oakman, D.D.S., M.D.* 28
Veneer Inlay - - - *By W. Clyde Davis, M.D., D.D.S.* 39
Indents - - - - - - - - - - - - 43
Announcements - - - - - - - - - - - 51

